# Epidemiology of Anatomical Variations in the Maxillary Sinus Drainage System and the Impact of Concha Bullosa

**DOI:** 10.31661/gmj.v13iSP1.3585

**Published:** 2024-12-29

**Authors:** Mohammadreza Shokuhifar, Hojatollah Yousefimanesh, Shiva Gandomi, Zeinab Soleimani, Shiva Gandomi, Hanie Hosseini Behbahani

**Affiliations:** ^1^ Department of Oral and Maxillofacial Radiology, School of Dentistry, Ahvaz Jundishapur University of Medical Sciences, Ahvaz, Iran; ^2^ Department of Periodontics, School of Dentistry, Ahvaz Jundishapur University of Medical Sciences, Ahvaz, Iran; ^3^ School of Dentistry, Ahvaz Jundishapur University of Medical Sciences, Ahvaz, Iran

**Keywords:** Cone-beam Computed Tomography, Paranasal Sinuses, Maxillary Sinus

## Abstract

**Background:**

The ostiomeatal complex (OMC) is an important anatomical structure which contains the maxillary ostium and infundibulum; sinus drainage is performed through this route. Concha bullosa, which is defined as pneumatization of the middle concha, may lead to drainage disorders and sinusitis following alterations in the OMC; hence, the purpose of this study was to investigate the effect of presence of concha bullosa on the infundibulum length and ostium height using cone-beam computed tomography (CBCT).

**Materials and Methods:**

This cross-sectional study evaluated 208 CBCT scans of 416 maxillary sinuses, which were retrospectively selected from the records available in the CBCT archives. Measurement of infundibulum length and maxillary ostium height were compared based on the presence of concha bullosa.

**Results:**

Out of 416 sinuses, 184 (44.2%) had concha bullosa. There was no significant relationship between concha bullosa and age or gender (P0.05). However, significant differences were observed in infundibulum length and ostium height between genders, with males having longer infundibulum lengths (right: 15.59 mm, left: 15.54 mm, total: 15.56 mm, P0.001) and higher ostium heights (right: 31.68 mm, left: 32.30 mm, total: 31.99 mm, P=0.001). Concha bullosa presence significantly affected infundibulum length and ostium height on the right side, with shorter lengths (11.83 mm vs. 13.47 mm, P=0.004) and higher heights (30.69 mm vs. 28.51 mm, P=0.001) in those with concha bullosa. No significant correlations were found between age and infundibulum length or ostium height (P0.1). Gender-specific differences were noted, with females showing significant impacts on the right side and males on both sides.

**Conclusion:**

In the present study, presence of concha bullosa shortened the infundibulum length and increased the ostium height. Also, the infundibulum length and ostium height were greater in males than females.

## Introduction

**Figure-1:**
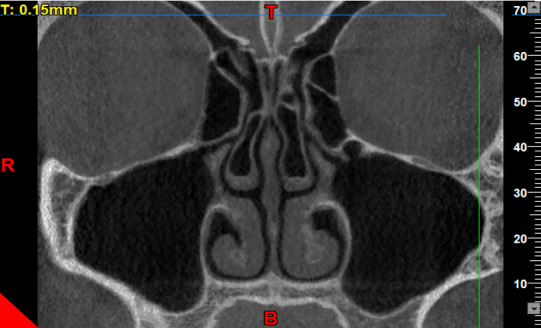
Figure 1. CBCT showing pneumatization of the middle concha. Note the pneumatization and radiolucency in the middle concha, which were detected by scrolling the coronal view.

**Figure-2:**
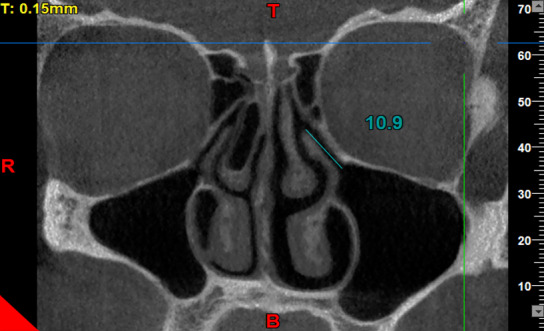
Figure 2. Measuring the infundibulum length

The maxillary sinuses are the first paranasal sinuses to develop, forming in the second month of fetal life. They are air-filled spaces in the maxillary bones on both sides of the face, expanding from a depression in the lateral wall of the nasal cavity [1][2].

The ostiomeatal complex (OMC) includes the maxillary ostium, ethmoidal infundibulum, middle nasal concha, uncinate process, ethmoid bulla, and hiatus semilunaris. It facilitates mucus drainage from the sinuses. In normal respiration, mucus flows through the ostium and infundibulum into the nasal cavity, with the uncinate process and ethmoid bulla defining the passage. Narrowing of this passage can lead to maxillary sinusitis by obstructing the sinus opening and increasing pressure inside the sinus cavity [3][4][5]. Therefore, it is reasonable to assume that the infundibulum length, the ostium height, and some other anatomical parameters of the OMC may affect the efficiency of mucus drainage, and may be associated with sinus pathologies [6].

Concha bullosa is defined as pneumatization of the middle turbinate, and is the most common anatomical variation of the OMC [7][8]. The causes of pneumatization are unclear; although trauma, nasal septal deviation, and mouth breathing are known as the predisposing factors to concha bullosa [9]. Since the middle turbinate is affected by the air flow, it becomes more pneumatized [8]. Concha bullosa has a negative effect on the paranasal sinus ventilation and mucus discharge of the middle meatus [3]. Also, the concha bullosa air cavity is covered with the same epithelium as the nasal cavity. These cells can cause inflammation and subsequent chronic disorders in the paranasal sinuses [10]. However, their potential effect on development of maxillary sinusitis has not been determined [3]. Computed tomography allows dental clinicians to identify and characterize pathological processes in the paranasal sinuses. Also, cone-beam computed tomography (CBCT) is often used to assess the sinus anatomy prior to dental implant placement, facilitating assessment of the maxillofacial region by providing data with high geometrical accuracy, isotropic voxel values, and short scanning time. It has a lower radiation dose, greater accuracy, and lower cost than computed tomography. Also, CBCT is used to assess the physiology and pathologies of the paranasal sinuses, including variations in the OMC [3].

As the current literature highlights the importance of the OMC in maxillary sinus drainage, yet fails to thoroughly investigate the impact of concha bullosa on the infundibulum length and ostium height, particularly in the Iranian population, we aimed to bridge this knowledge gap by examining the relationship between concha bullosa and these anatomical parameters using CBCT in a sample of Iranian patients. Moreover, our study novelly explores the effect of concha bullosa on the maxillary sinus drainage system in this population, providing new insights into the potential mechanisms underlying maxillary sinusitis, which have not been fully elucidated in previous studies.

## Materials and Methods

This retrospective cross-sectional study evaluated CBCT scans of 208 patients referred to a private maxillofacial radiology clinic during 2020-2022. The inclusion criteria were optimal quality of images, available information about the age and gender of patients, and complete visualization of the maxillary sinuses and the nasal cavity on the CBCT scans. Patients with a history of surgery, implant placement, bone graft, pathologies, mid-facial trauma, sinusitis, or complete maxillary sinus and ostium obstruction, and patients under 15 years of age were excluded from the study. Most CBCT scans in this radiology department were performed for dental implant planning or diagnosis of maxillofacial pathologies.

All CBCT scans had been taken with NewTom Giano CBCT scanner (QR, Verona, Italy) with 11 x 8 cm field of view, high resolution, and exposure settings of 90 kVp tube potential, and 9 seconds time, saved in NNT Viewer version 10.1 and 11, and were available in the archives. The CBCT scans were viewed in a semi-dark room on a 14-inch LED flat-screen monitor (Asus) with 1920 x 1080 resolution. The images were reconstructed such that the hard palate was parallel to the horizon, and the sagittal plane was perpendicular to the horizon. The MPR feature of the NNT Viewer software was used to make the necessary measurements.

**Figure-3:**
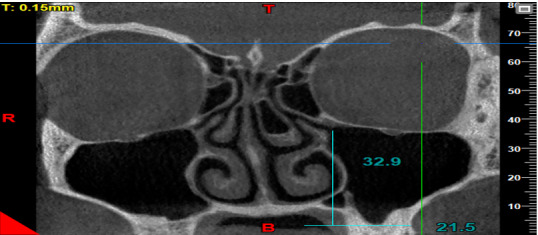
Figure 3. Measuring the ostium height

### Sample Size

The sample size was calculated using the following formula considering α=0.05, P=0.389 and d=0.07.


n = \frac{\left(Z_{1-\frac{\alpha}{2}}\right)^{2} \, \left(p(1-p)\right)}{d^{2}}

### Measurements

The axial, coronal, and sagittal views were used to assess the presence/absence of concha bullosa (Figure-1), and to measure the infundibulum length and ostium height (the most reliable and commonly used view to identify concha bullosa and make the measurements is the coronal view). The reviewed images visualized both the maxillary sinuses and the nasal cavity. Images with poor quality and those not visualizing the sinus floor, showing complete obstruction of the ostium, or lacking the middle concha were excluded. The patients’ age and gender, presence/absence of right and left concha, infundibulum length, and right and left ostium height were all recorded. The CBCT scans were evaluated by a board-certified oral and maxillofacial radiologist with 20 years of clinical experience. Subjectivity of the measurements was a source of bias in this study. To verify the reliability of the measurements, the intra-examiner reliability was calculated by repeating the measurements after a 3-month interval. The intraclass correlation coefficient was calculated to be 95%, indicating excellent intra-examiner reliability. To measure the dimensions of the drainage system, the infundibulum length and the ostium height were measured on both sides. For this purpose, the coronal view was scrolled to select the cross-section in which the uncinate process and the path of infundibulum and ostium were clearly visible, had an open path, and the largest values of the length and height were measured. The maximum length and height could be found on one or two different cross-sections. The infundibulum length was considered as the distance between the center of the ostium and the highest point of the uncinate process [3] (Figure-2). The ostium height was considered as the distance between the center of the ostium and the lowest point of the floor of the maxillary sinus [3]. To measure this variable, a line was drawn tangent to the lowest part of the sinus floor and parallel to the horizon. Next, a line was drawn perpendicular to the above-mentioned tangent line from the center of the ostium, and the obtained height was recorded (Figure-3).

### Statistics Analysis

The data were analyzed using SPSS version 22 (SPSS Inc., Chicago, IL, USA) by independent samples t test, Chi-square test, Mann-Whitney U test, and the Spearman's correlation test at 0.05 level of significance.

## Results

**Table:** Table1. Presence/Absence of Concha Bullosa According to Age

^Concha bullosa ^		Mean	SD	P-value**
Right side	Absent	29	10.72	0.503
	Present	27.55	7.2	
Left side	Absent	27.6	7.96	0.704
	Present	28.06	8.44	
Total	Absent	28.93	9.28	0.458
	Present	27.81	7.84	

**Table:** Table2. Presence/Absence of Concha Bullosa According to Gender

	^Concha bullosa ^	^Gender^ ^n (%)^		^P-value*^
		Female	Male	
^Right side ^	^Absent^	^89(56.0)^	^29(59.2)^	^0.743^
	^Present^	^70(44.0)^	^20(40.8)^
^Left side ^	^Absent^	^88(55.3)^	^26(53.1)^	^0.870^
	^Present^	^71(44.7)^	^23(46.9)^
^Total^	^Absent^	^177(55.7)^	^55(56.1)^	^0.515^
	^Present^	^141(44.3)^	^43(43.9)^

**Table:** Table3. Mean Values and Standard Deviation of Infundibulum Length and Ostium Height in Millimeters According to Gender

Variable	Gender	Mean	Standard deviation	P-value*
Infundibulum length (right)	Female	11.99	3.58	<0.001
	Male	15.59	3.61	
Infundibulum length (left)	Female	12.63	3.2	<0.001
	Male	15.54	4.31	
Infundibulum length (total)	Female	12.26	3.41	<0.001
	Male	15.56	3.95	
Ostium height (right)	Female	28.77	4.22	0.001
	Male	31.68	5.5	
Ostium height (left)	Female	29.05	4.3	0.001
	Male	32.3	6.07	
Ostium height (total)	Female	28.91	4.25	0.001
	Male	31.99	5.77	

**Table:** Table4. Mean Values and Standard Deviation of Infundibulum Length and Ostium Height in Millimeters According to Presence/Absence of Concha Bullosa

	concha bullosa	Infundibulum length		P-value	Ostium height		P-value
		Mean	SD		Mean	SD	
Right side	Absent	13.47	4.04	0.004	28.51	4.95	0.001
	Present	11.83	3.54		30.69	4.07	
Left side	Absent	13.5	3.61	0.321	29.26	4.99	0.076
	Present	13.09	3.81		30.48	4.85	
Total	Absent	13.49	3.83	0.007	28.88	4.97	<0.001
	Present	12.48	3.73		30.58	4.47	

**Table:** Table5. Relationship of Infundibulum Length and Ostium Height with Age

^Variable^	^Correlation Coefficient^	^P-value*^
^Infundibulum length (right)^	^0.107^	^0.132^
^Infundibulum length (left)^	^0.049^	^0.485^
^Infundibulum length (total)^	^0.079^	^0.106^
^Ostium height(right)^	^0.035^	^0.615^
^Ostium height (left)^	^0.002^	^0.982^
^Ostium height (total)^	^0.019^	^0.702^
^*^Spearman's rank correlation coefficient

The study evaluated CBCT images of 208 maxillary sinuses from patients with a mean age of 28.43 years, with a significant female predominance (76.4%, n=159) compared to males (23.6%, n=49). Out of 416 sinuses, 184 (44.2%) had concha bullosa. Table-1 presents the presence or absence of concha bullosa according to age. For the right side, the mean age for patients without concha bullosa was 29 years (SD=10.72), while for those with concha bullosa, it was 27.55 years (SD=7.2), with a P-value of 0.503 indicating no statistically significant difference. On the left side, the mean age for patients without concha bullosa was 27.6 years (SD=7.96), and for those with concha bullosa, it was 28.06 years (SD=8.44), with a P-value of 0.704, also indicating no significant difference. Overall, the mean age for patients without concha bullosa was 28.93 years (SD=9.28), and for those with concha bullosa, it was 27.81 years (SD=7.84), with a P-value of 0.458, further confirming no significant age-related differences in the presence of concha bullosa. Table-2 summarizes the presence or absence of concha bullosa according to gender. For the right side, 56.0% (n=89) of females and 59.2% (n=29) of males had concha bullosa absent, with a P-value of 0.743, indicating no significant gender difference. On the left side, 55.3% (n=88) of females and 53.1% (n=26) of males had concha bullosa absent, with a P-value of 0.870, also showing no significant difference. Overall, 55.7% (n=177) of females and 56.1% (n=55) of males had concha bullosa absent, with a P-value of 0.515, further confirming no significant gender-related differences in the presence or absence of concha bullosa.

Table-3 shows significant differences in both infundibulum length and ostium height between males and females. For infundibulum length, the mean values for females were significantly lower than for males on both the right and left sides, as well as overall (P<0.001). Similarly, for ostium height, the mean values for females were significantly lower than for males on both the right and left sides, as well as overall (P=0.001).

Table-4 presents the mean values and standard deviations of infundibulum length and ostium height in millimeters, according to the presence or absence of concha bullosa. For the right side, the mean infundibulum length was 13.47 mm (SD=4.04) in the absence of concha bullosa and 11.83 mm (SD=3.54) in its presence, with a significant difference (P=0.004). The mean ostium height was 28.51 mm (SD=4.95) in the absence of concha bullosa and 30.69 mm (SD=4.07) in its presence, also showing a significant difference (P = 0.001). For the left side, the mean infundibulum length was 13.50 mm (SD=3.61) in the absence of concha bullosa and 13.09 mm (SD=3.81) in its presence, with no significant difference (P=0.321). The mean ostium height was 29.26 mm (SD=4.99) in the absence of concha bullosa and 30.48 mm (SD=4.85) in its presence, with no significant difference (P = 0.076). Overall, the mean infundibulum length was 13.49 mm (SD=3.83) in the absence of concha bullosa and 12.48 mm (SD=3.73) in its presence, with a significant difference (P=0.007). The mean ostium height was 28.88 mm (SD=4.97) in the absence of concha bullosa and 30.58 mm (SD=4.47) in its presence, with a highly significant difference (P<0.001). Table-5 presents the relationship between infundibulum length and ostium height with age, using Spearman's rank correlation coefficient. For infundibulum length, the correlation coefficients were 0.107 (P=0.132) for the right side, 0.049 (P=0.485) for the left side, and 0.079 (P=0.106) for the total, indicating no statistically significant correlations with age. For ostium height, the correlation coefficients were 0.035 (P=0.615) for the right side, 0.002 (P=0.982) for the left side, and 0.019 (P=0.702) for the total, also showing no significant correlations with age. Table-6 compares the mean infundibulum length and ostium height in millimeters according to the presence or absence of concha bullosa in the right and left sides for both males and females. For females, on the right side, the mean infundibulum length was significantly longer in those with concha bullosa (12.52 mm, SD=3.66) compared to those without (11.09 mm, SD=3.34; P=0.006). Similarly, the mean ostium height was significantly lower in those with concha bullosa (27.93 mm, SD=4.61) compared to those without (29.83 mm, SD=3.41; P=0.003). On the left side, there were no significant differences in infundibulum length (P=0.659) or ostium height (P=0.698) between those with and without concha bullosa. For males, on the right side, the mean infundibulum length was not significantly different (P=0.061), but the mean ostium height was significantly lower in those with concha bullosa (30.28 mm, SD=5.6) compared to those without (33.70 mm, SD=4.81; P=0.027). On the left side, both the mean infundibulum length (P=0.029) and ostium height (P=0.017) were significantly lower in those with concha bullosa (16.80 mm, SD=3.79 and 30.38 mm, SD=6.12, respectively) compared to those without (14.12 mm, SD=4.5 and 34.46 mm, SD=5.35, respectively).

## Discussion

**Table:** Table6. Comparison of the Mean Infundibulum Length and Ostium Height in Millimeters According to Presence/Absence of Concha Bullosa in the Right and Left Sides in Males and Females

		concha bullosa	Infundibulum length			Ostium height		
			mean	SD	P	mean	SD	P
Female	right side	Present	12.52	3.66	0.006	27.93	4.61	0.003
		Absent	11.09	3.34		29.83	3.41	
	left side	Present	12.53	2.93	0.659	28.93	4.6	0.698
		Absent	12.76	3.54		29.19	3.92	
Male	right side	Present	16.39	3.81	0.061	30.28	5.6	0.027
	Absent		14.43	3		33.7	4.81	
	left side	Present	16.8	3.79	0.029	30.38	6.12	0.017
		Absent	14.12	4.5		34.46	5.35	

^*^Independent sample t-test; **SD:** Standard deviation

The purpose of this study was to investigate the relationship of the presence of concha bullosa with the infundibulum length and ostium height using CBCT.The results were analyzed in the following sections: In the present study, 44.2% of the patients had concha bullosa. Also, most of the cases of concha bullosa (48.4%) were bilateral, similar to the study by Kar et al [11].

In contrast to the present study, Al-Rawi et al. [9] showed that 20.7% of patients had unilateral concha bullosa, and 16.6% had bilateral concha bullosa. This difference may be due to differences in sample size, ethnicity, race, CBCT scanners, and software programs used for the assessments. The infundibulum length was shorter, and the ostium height was greater in presence of concha bullosa,, and this relationship was significant. However, Akay et al. [3] showed that there was no significant relationship between the infundibulum length and ostium height in presence of concha bullosa, which was inconsistent with the results of the present study. This difference can be due to differences in race, type of imaging modality, and software programs.

Shin et al. [12] showed longer and narrower infudibulum in presence of a larger concha bullosa, which was in agreement with the present results. De Carvalho et al. [4] demonstrated that the smaller the ostium height and the shorter the infundibulum length, the easier it would be to drain the sinus; resultantly, the probability of accumulation of secretions and subsequent sinusitis would decrease. The current study found that presence of concha bullosa shortened the infadibulum length (a positive effect reducing the risk of development of sinusitis), but increased the ostium height (a negative effect leading to developiment of sinusitis). However, the literature in this regard is controversial such that Azila et al. [13] did not find a significant relationship between the presence of concha bullosa and sinusitis.

The present results in this regard were consistent with the findings of Akay et al, [3], de Carvalho et al, [4] and Vaddi et al [14]. Akay et al, [3] and Mohammed et al, [15] also concluded that the difference in infundibulum length between males and females was not significant, and the relationship between the infundibulum length and gender was not significant either, which was contrary to the present findings. The present study found a significant relationship in this regard which was in line with the results of de Carvalho et al, [4] who showed that the length of the infudibulum was greater in males than females. Such differences can be due to genetic differences of different study populations.

The present study indicated that there was no significant relationship between the infundibulum length and ostium height with age, which was in line with studies conducted by Mohammed et al, [15] and Shetty et al [16].

Tomblinson et al, [17] and Akay et al. [3] found that there was no significant relationship between presence/absence of concha bullosa and age or gender. Also, Koo et al. [18] did not find a significant association between age and concha bullosa, and Al-Rawi et al. [9] did not find a significant relationship between gender and concha bullosa, which were in line with the results of the present study.

the infundibulum length was 13.49 mm (without concha bullosa) and 12.48 mm (with concha bullosa), with a significant difference (P=0.007). The ostium height was 28.88 mm (without concha bullosa) and 30.58 mm (with concha bullosa), with a highly significant difference (P<0.001). There is a discrepancy between our study and the Akay et al. [ 19] study regarding the effect of concha bullosa on the maxillary sinus drainage system. The study by Akay et al. evaluated the correlation between the dimensions of the maxillary sinus drainage system and anatomical variations using CBCT, finding that ostium height was significantly greater in males and correlated with the presence of maxillary sinus septa, but other variations like nasal septal deviation, concha bullosa, and Haller cells did not significantly affect the dimensions of the maxillary sinus drainage system. Our study, which involved an Iranian population, may have observed different anatomical variations and effects due to genetic and ethnic factors. Populations from different geographic regions and ethnic backgrounds can exhibit significant variations in craniofacial and sinus anatomy. For example, the prevalence and impact of concha bullosa might be more pronounced in the Iranian population due to genetic predispositions specific to that region. The study by Generato Sogono and Gozun Songco found that in Filipino patients, maxillary sinusitis was associated with male gender and larger infundibular size, but not with nasal septal deviation or concha bullosa [20].

Similar to our study, some studies have shown that anatomical variations, such as concha bullosa, can affect the dimensions of the maxillary sinus drainage system, including the length of the infundibulum. For instance, a study by Ono et al. (2011) found that the presence of concha bullosa influenced sinus opacification in both allergic and non-allergic rhinitis groups [21].

## Conclution

The findings of the present study showed that the infundibulum length and the ostium height were greater in males than in females and this difference was significant. Also, the relationship between the infundibulum length and ostium height with age, and the relationship of concha bullosa with age and gender were not significant.

## Conflict of Interest

None.
